# Electrophysiological properties and structural prediction of the SARS-CoV-2 viroprotein E

**DOI:** 10.3389/fmolb.2024.1334819

**Published:** 2024-03-28

**Authors:** Salvatore Antonio Maria Cubisino, Stefan Milenkovic, Stefano Conti-Nibali, Nicolò Musso, Paolo Bonacci, Vito De Pinto, Matteo Ceccarelli, Simona Reina

**Affiliations:** ^1^ Department of Biomedical and Biotechnological Sciences, University of Catania, Catania, Italy; ^2^ Department of Physics, University of Cagliari, Cagliari, Italy; ^3^ We.MitoBiotech S.R.L, Catania, Italy

**Keywords:** pore-forming peptide, molecular modelling and simulations, electrophysiology, protein arrangement, ionic conductance, envelope (E) protein, single channel recordings, calcium conduction

## Abstract

COVID-19, the infectious disease caused by the most recently discovered coronavirus SARS- CoV-2, has caused millions of sick people and thousands of deaths all over the world. The viral positive-sense single-stranded RNA encodes 31 proteins among which the spike (S) is undoubtedly the best known. Recently, protein E has been reputed as a potential pharmacological target as well. It is essential for the assembly and release of the virions in the cell. Literature describes protein E as a voltage-dependent channel with preference towards monovalent cations whose intracellular expression, though, alters Ca^2+^ homeostasis and promotes the activation of the proinflammatory cascades. Due to the extremely high sequence identity of SARS-CoV-2 protein E (E-2) with the previously characterized E-1 (i.e., protein E from SARS-CoV) many data obtained for E-1 were simply adapted to the other. Recent solid state NMR structure revealed that the transmembrane domain (TMD) of E-2 self-assembles into a homo-pentamer, albeit the oligomeric status has not been validated with the full-length protein. Prompted by the lack of a common agreement on the proper structural and functional features of E-2, we investigated the specific mechanism/s of pore-gating and the detailed molecular structure of the most cryptic protein of SARS-CoV-2 by means of MD simulations of the E-2 structure and by expressing, refolding and analyzing the electrophysiological activity of the transmembrane moiety of the protein E-2, in its full length. Our results show a clear agreement between experimental and predictive studies and foresee a mechanism of activity based on Ca^2+^ affinity.

## Graphical Abstract

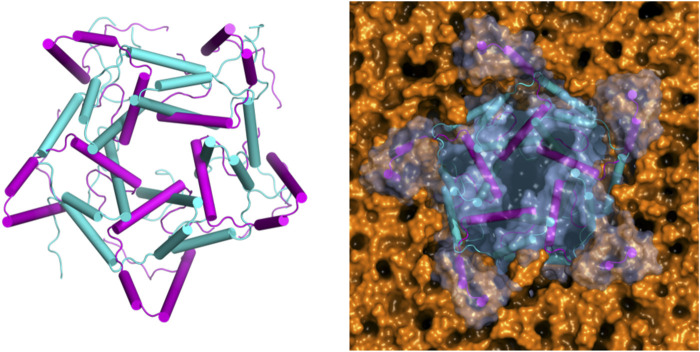

## Introduction

COVID-19 pandemic was due to severe acute respiratory syndrome coronavirus 2 (SARS- CoV-2), a potentially fatal respiratory disease. SARS-CoV-2, discovered in December 2019 in Wuhan (China), is a single-stranded RNA virus that is not yet fully understood and that continues to produce new variants and to rapidly spread worldwide (Hu et al., 2021). It contains 14 open reading frames (ORFs) (Gordon et al., 2020) encoding 31 proteins organized as follows: i) two large polyproteins (ORF1a and ORF1ab) whose proteolytic cleavage leads to 16 non-structural proteins (nsp1- 16) implicated in genome replication and early transcription regulation (Perlman and Netland, 2009); ii) four structural proteins called spike (S), membrane (M), envelope (E) and nucleocapsid (N), which are considered key targets for the development of antiviral drugs and assemble with the positive-sense single-stranded RNA genome to form SARS-CoV-2 virions (Li, 2016; Tortorici and Veesler, 2019; Bai et al., 2021; Zhang et al., 2022); iii) eleven accessory proteins (ORF3a, ORF3b, ORF3c, ORF3d, ORF6, ORF7a, ORF7b, ORF8, ORF9b, ORF9c and ORF10) that have been proposed to mediate virus-host interactions during infection (Vkovski et al., 2021). Whilst the spike, responsible for virus binding to its surface receptor on target cells, has undoubtedly focused many efforts for therapies and vaccines development, the envelope protein is emerging as a worthy candidate as well. It is the smallest among the structural proteins and it is highly conserved in different viral subtypes, although its role in viral invasion, replication and release has not been sufficiently elucidated. Jointly with the membrane protein M, E is required for the assembly of the virion envelope (DeDiego et al., 2007). Accordingly, the lack of E significantly reduces viral titer and cripples viral maturation (Kuo and Masters, 2003; DeDiego et al., 2007). Mutation of protein E sequence induces apoptosis (Xia et al., 2021) and, in addition, co-expression of E and M promotes spike re-localization to the endoplasmic reticulum‐Golgi intermediate compartment (ERGIC) and Golgi (Boson et al., 2021). Noteworthy, the envelope protein is highly expressed during the CoV replication cycle but, in the end, it is scarcely inserted into virions as it is mainly located in internal membranes (ER, ERGIC and Golgi compartments) responsible for virus assembly and budding (McClenaghan et al., 2020; Sarkar and Saha, 2020).

From a structural point of view, E is an integral membrane protein of 75–109 amino acids with a short hydrophilic N-terminus that protrudes towards the lumen, followed by a large hydrophobic transmembrane domain (TMD) of 25aa and a long hydrophilic C-terminus with a cytosolic orientation (Schoeman and Fielding, 2019). Thanks to its position, the C-terminus allows E to interact with other viral and host proteins. In particular, intraviral interactions between E and M, which have been demonstrated also within host cells (Chen et al., 2021), involve the C-terminal domains of both proteins. Moreover, the triple cysteine motif (C40, C43, and C44) in E protein has been proposed to associate with the cysteine-rich C-terminal region of S protein whereas the C-terminal DLLV sequence has been identified as a PDZ-binding motif that binds to the host cell polarity signaling protein PALS1 and enhances the destruction of epithelial integrity, consequently fueling the inflammatory processes (Schoeman and Fielding, 2019). The protein structure, however, is controversial: a very recent solid state NMR structure revealed the TMD of SARS-CoV-2 E protein (E-2) self-assembles into a homo-pentamer (Surya et al., 2018), although the oligomeric status has not been validated by structural data of the full-length protein.

Computational studies on monomeric E-2 (Kuzmin et al., 2022) showed that the transmembrane α-helix is tilted in the membrane, while the C-terminal α-helices are free to move around it. Furthermore, the expression of E-2 induces membrane curvature, both in monomeric and oligomeric forms (Kuzmin et al., 2022; Mehregan et al., 2022). Due to the high sequence identity between SARS-CoV and SARS-CoV-2 protein E (∼95%), several literature data regarding E-2 exploits the structural similarity with the previously characterized E-1 and mainly utilizes synthetic peptides corresponding to the TMD (Verdiá-Báguena et al., 2021). The transmembrane domain of SARS‐CoV‐2 E protein exhibits a completely conserved amino‐acid sequence (residues 15-37) compared to that of SARS‐CoV‐1 E protein. Similarly to other virus-encoded transmembrane proteins called viroporins, the SARS-CoV E protein forms pores that facilitate ion transport across cell membranes. Bilayer (Verdiá-Báguena et al., 2021; Xia et al., 2021) and patch-clamp recordings (Cabrera-Garcia et al., 2021; Mehregan et al., 2022) as well as MD simulation (Cao et al., 2020) of the full-length E-2 suggest it is a voltage-dependent hydrophobic channel with monovalent cation-selectivity. Still, its intracellular expression increases intracellular-Golgi pH and alters Ca^2+^ homeostasis (Nieto-Torres et al., 2015) eventually boosting the activation of a pro-inflammatory cascade with worsening of several respiratory symptoms.

The proposed NMR oligomer is composed by 5 units, where the long transmembrane (TM) helices create a tight bundle with a supposed central water pore. However, this model does not show the possibility to form a water-pore stable enough for the transport of ions (Kuzmin et al., 2022; Mehregan et al., 2022), compatible with electrophysiology experiments. Starting from the proposed In this arrangement of the open state, the helix bundle collapses, regardless of whether enhanced sampling techniques or coarse-grained simulations are employed. Only by keeping strong constraints on the initial structure, the central water-pore remains stable. Further, helix bundles designed rationally showed the same paradigm: the structure obtained from X-ray diffraction does not correspond to a stable water pore able to conduct, whilst some other different arrangements of helices can provide ionic current and selectivity in agreement with electrophysiological data (Scott et al., 2021).

Knowing it might exist an experimental structure different from the functional one, we investigate the structure and channel activity of E-2 combining electrophysiology experiments and molecular simulations on the complete 1-75 peptides. Apart from gathering key information of the structure and functioning of the E peptide, the topic of pore-forming peptide _systems_ is challenging. Recent investigations showed how it is possible to use single channel measurements to rationalize mutagenesis experiments, searching for specific properties (selectivity) tailored on technological applications (Krishnan et al., 2022; Puthumadathil et al., 2022).

## Results

### PLB measurements demonstrates E2 inserts into artificial membrane as a multimer

**FIGURE 1:**
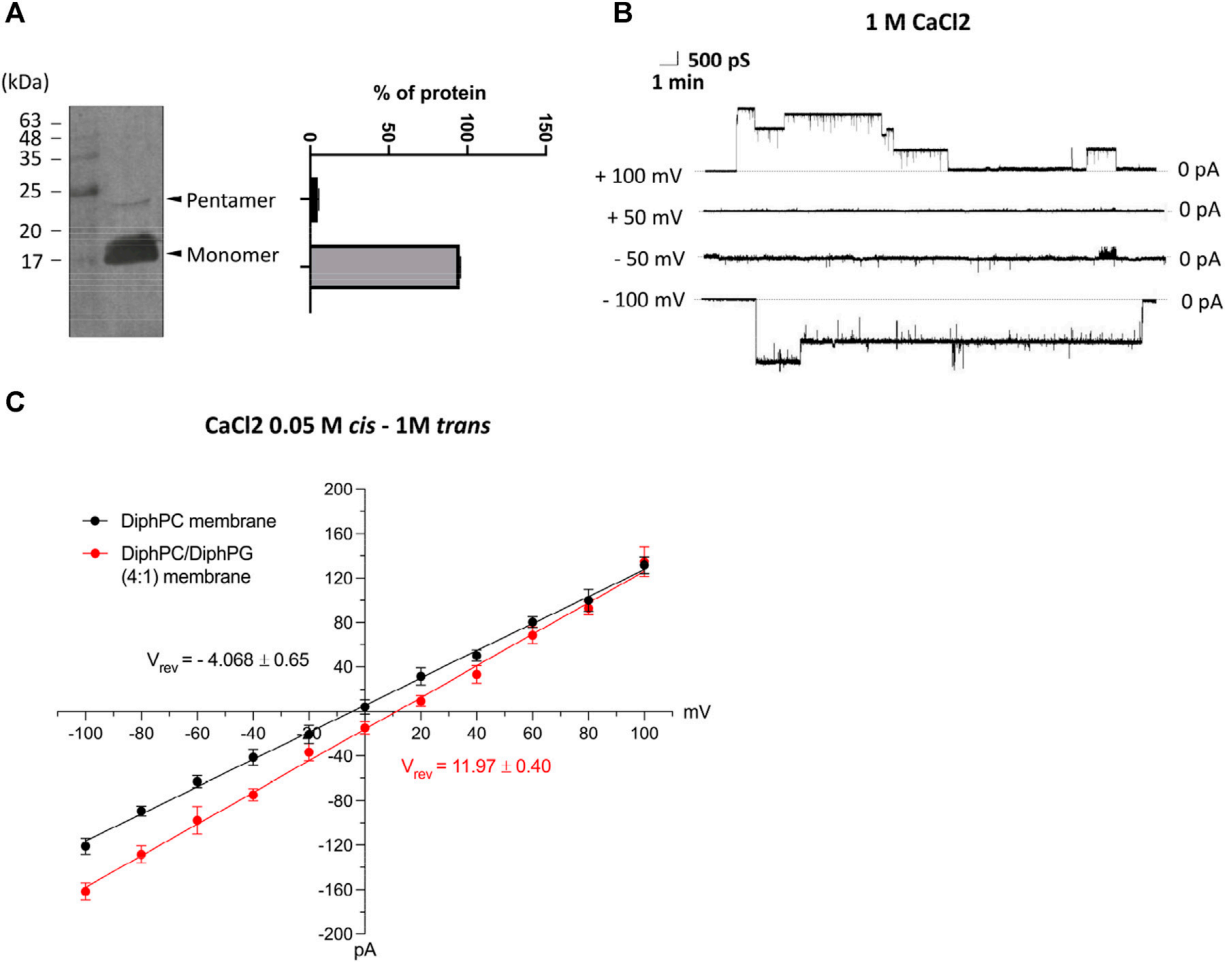
**(A)** Western blot analysis of SARS-CoV-2 protein E with anti-6xHis and quantification of monomeric and oligomeric forms. **(B)** Representative current trace of E-2 after reconstitution in lipid bilayers made of 1% DiphPC recorded at ± 100 mV in 1M CaCl_2_. **(C)** I/V plot of ion selectivity measurements performed in 1 M (*trans*) to 0.05 M (*cis*) CaCl_2_ solution in different lipid bilayers (DiphPC and DiphPC/DiphPG 4:1) upon application of a triangular voltage ramp of 0 ± 100 mV (n ≥ 3). All error bars are SEM.

The sequence template for the *in vitro* expression of E-2 protein was obtained from a viral specimen, selected as described in Methods. Then, after cloning of the cDNA replica into a plasmid, the small protein was expressed with an 6xHis-tag to accelerate its purification. The expression of recombinant E-2 was checked by western blot analysis. As shown in Figure 1, two protein bands were detected by anti-6xHisTag Ab at approximately 25 kDa and 17 kDa, presumably corresponding to the oligomeric and monomeric states, respectively, as already reported in Xia B. et al. (Xia et al., 2021) The expression and purification procedure was repeated twice with identical results. This preparation was evaluated for membrane insertion and production of electrophysiological recordings in planar artificial bilayers. The solution bathing the two sides of the membrane was a symmetrical 1M CaCl_2_ solution (pH 6.0). Upon application of ±100 mV the experiment revealed E-2 inserts as channels of ∼2 ± 0.4 nS that quickly move to low-conducting or closed states of approximately 700–800 pS (Figure 1B). No insertion was detectable at lower membrane potentials (±50 mV). The pore preference towards anions or cations (ion selectivity) was calculated under asymmetrical bi-ionic conditions. The fully open state of E-2, reconstituted in a 1% DiphPC membrane immersed in 1.0 M (*trans*) to 0.05 M (*cis*) asymmetric CaCl_2_ gradient and evaluated in the range of 0 to ±100 mV, exhibited a reversal potential (Ψrev) of −4.068 ± 0.65 mV, indicative of slightly anion selectivity (Figure 1C). The corresponding permeability ratio between Cl– and Ca2+ (P_cl_/P_ca_), calculated according to the Goldman-Hodgkin-Katz equation adapted for the evaluation of the calcium ion (Alvarez and Latorre, 2017), was 2.6. Under the same experimental conditions, the presence of a negatively charged PLB membrane consisting of DiphPC and DiphPG in ratio 4:1, increased the passage rate of cations through the E-2 pore with a Ψrev of +11.47 ± 0.40 mV and a P_cl_/P_ca_ of 0.9 (Figure 1C).

### Molecular dynamics simulations of the E2 model a suggest a new pentameric arrangement

**FIGURE 2:**
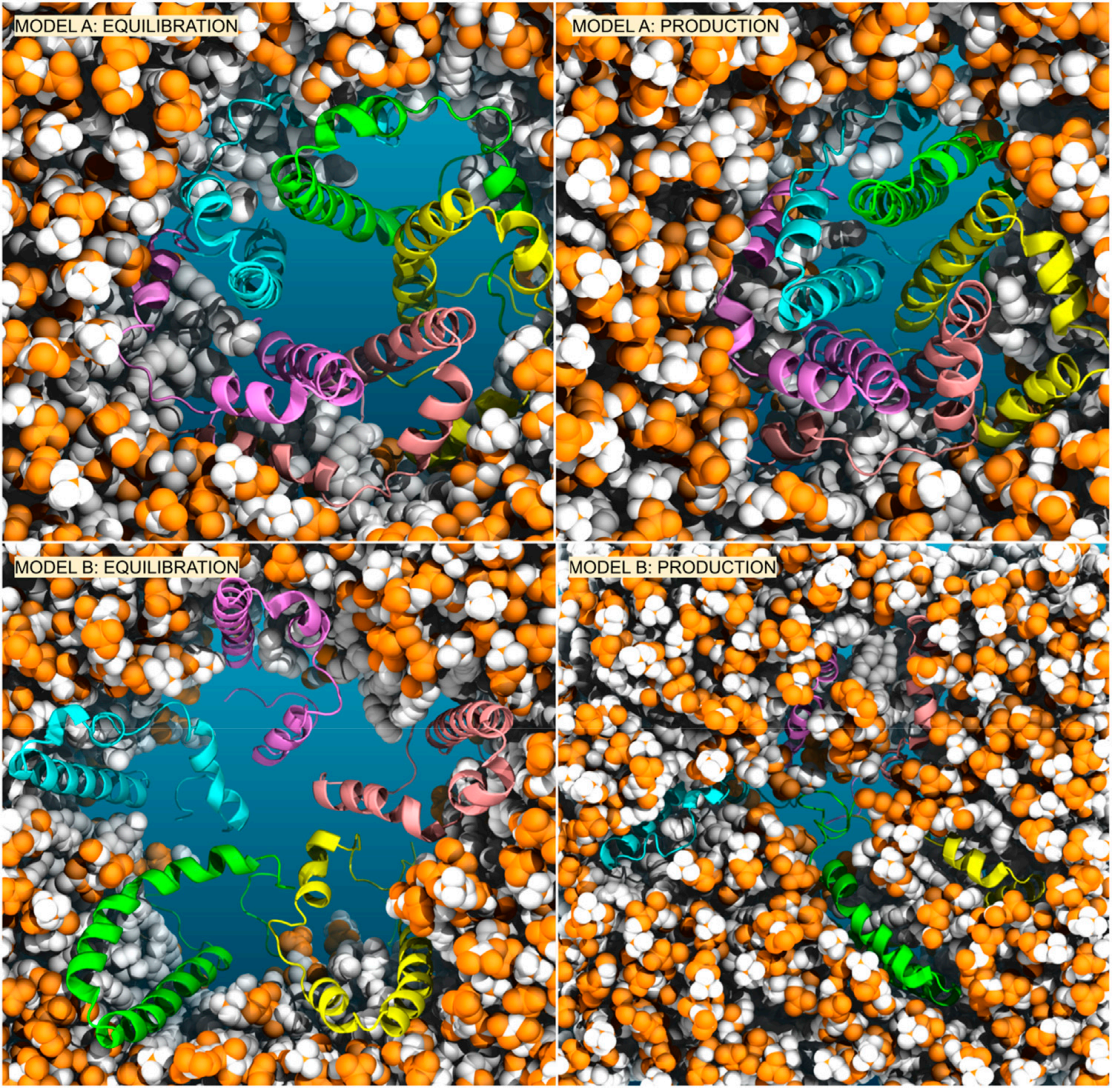
Comparison of Model A (top) *versus* Model B (bottom) embedded in the membrane. We reported the conformers after equilibration with restraints on peptides (left) and after 1 us of production simulations (right).

We conducted simulations of the proposed E-2 pentameric model, starting from the NMR structure (referred to here as Model A). We embedded it in a pre-equilibrated bilayer using the CHARMM-GUI tool and we solvated the complex with either 150 mM KCl or 50 mM KCl +50 mM CaCl_2_. To obtain the full-length E-2 peptide, the missing residues were filled using the Modeller (Webb and Sali, 2016) software, as described in the Supplementary Information. The initial pentameric structure of Model A is depicted in Figure 2 (top-left) embedded in the membrane (see also Supplementary Figure S1), with the central pore solvated and water present at the periphery near the amphiphilic helices (Supplementary Figure S2). Surprisingly, when we subjected the sequence to simulation, after equilibration and the release of constraints, the assumed pentameric structure, with the longest helices forming a bundle, collapses into a closed configuration, expelling water from interior (Figure 2-top-right and Supplementary Figure S1/S2). This collapsed structure is consistently observed even when using different membrane compositions, enhanced sampling methods, or extended equilibration with constraints, in line with recent data (Mehregan et al., 2022).

**FIGURE 3:**
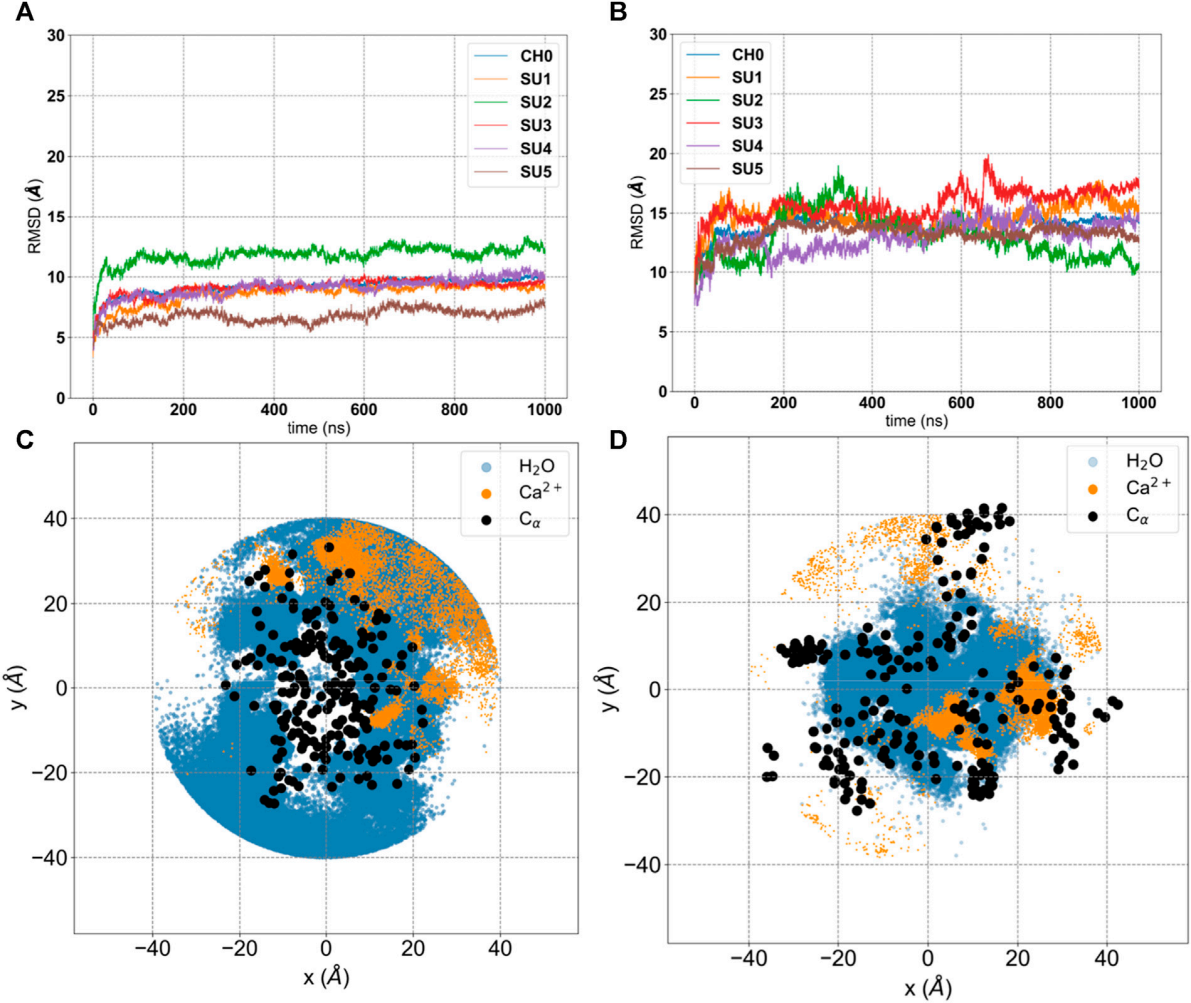
Analysis of 1 μs production run trajectory (50 mM KCl +50 mM CaCl_2_) of E-2 Model A *versus* E-2 Model **(B)**. **(A, B)** Root-Mean-Square Deviation (RMSD) of each subunit (SU 1-5) and the entire structure (CH0) with respect to the initial conformation. **(C, D)** projection on the XY plane of coordinates extracted from the trajectory for water molecules (blue), calcium ions (orange) and c-α of hydrophobic residues (black).

**FIGURE 4:**
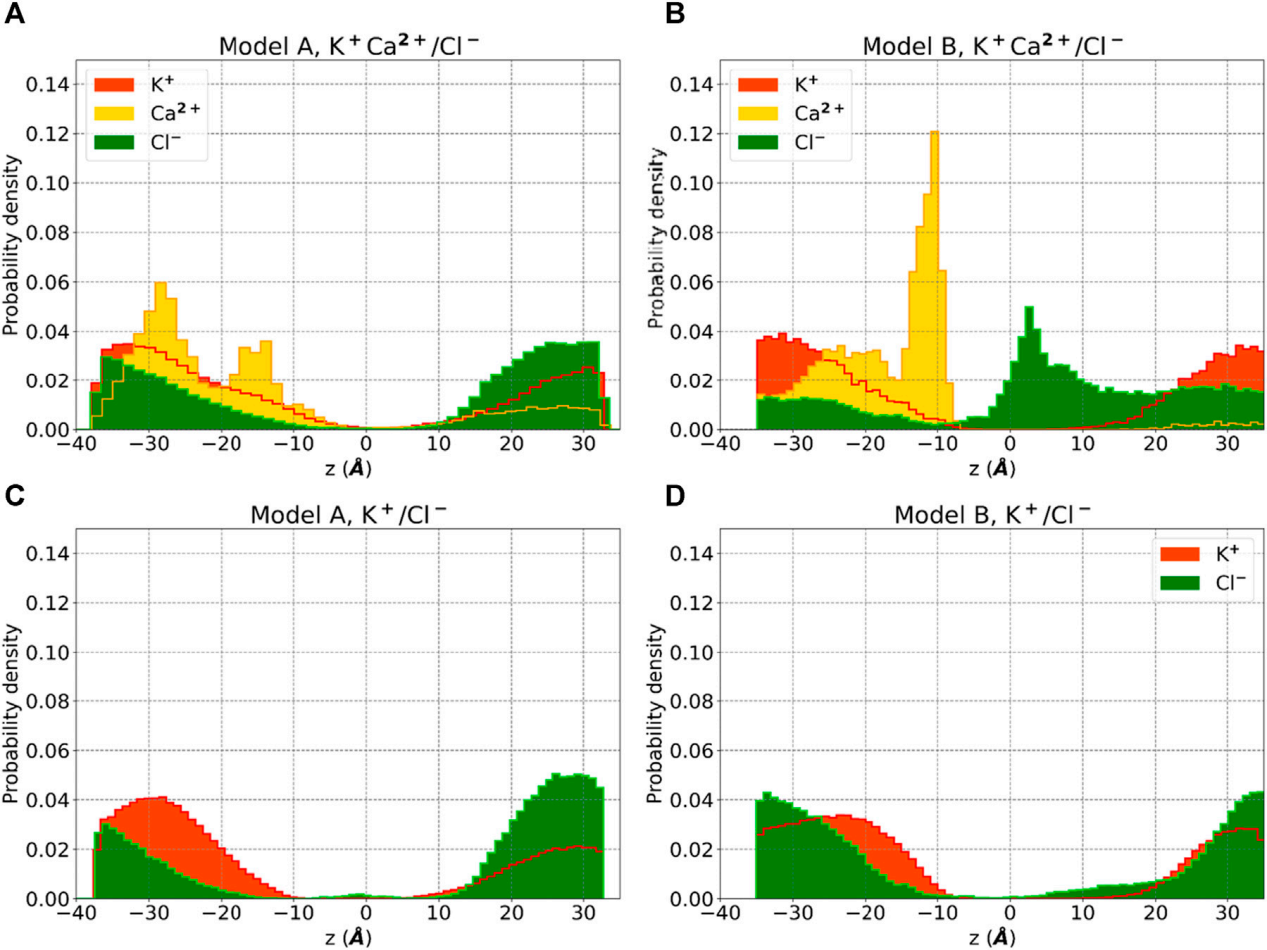
Ion density for Model A and Model B obtained by standard MD simulations using 0.05M KCl +0.05M CaCl2 in buffer solution (**A, B**) and 0.15 M KCl (**C, D**). Probability densities of potassium (red), chloride (green) and calcium (yellow) are reported along the z-axis of the membrane.

Analysis of our standard MD trajectories revealed that, after the initial rearrangement leading to the collapse, the oligomer remains stable on the microsecond timescale. The RMSD (Figure 3A), calculated for each unit after fitting to the entire pentamer, indicates that only one subunit (SU2) deviates from the overall oligomer, suggesting a potential tetrameric arrangement of the bundle. To illustrate the absence of a water-filled pore, in Figure 3C, we overlaid the water density, calcium density, and positions of hydrophobic residues (c-alpha coordinates) on the XY plane. Notably, the central region is occupied by hydrophobic residues, and water is absent. Despite the disappearance of the central water pore after the collapse, water persists in the membrane around the externally positioned short helices. Interestingly, calcium ions tend to accumulate where water is present, primarily on the supposed luminal side, with two prominent peaks (see Figure 4); neither potassium or chloride ions occupy the central region from [-10,10] Å.

To assess ion transport, we applied an external electric field of +100 mV to Model A for 2 µs No ions movement was observed, and even when we increased the voltage to +400 mV, no ion current appeared. In conclusion, the arrangement of the pentamer described by Model A does not permit ion transport on the microsecond timescale.

Our analysis of Model A simulations revealed the presence of water within the membrane, forming lateral discontinuous water paths, coinciding with the positions of the short amphipathic helices, though insufficient for ion transport. To explore an alternative arrangement of peptides, we oriented them in the opposite direction relative to the membrane axis (see in Supplementary Figure S3 the scheme of both models and the rotation of 180^o^ applied independently to each peptide and able to transform Model A in Model B), with the short amphipathic helices facing the interior and the long hydrophobic helices toward the exterior, in direct contact with the membrane, see Figure 2-bottom and Supplementary Figure S4. This arrangement, here referred to as Model B, would encourage the aggregation of the short amphipathic helices and potentially the formation of a larger and stable central hydrophilic pore. This arrangement, which has been previously suggested by Schoeman et al. (Schoeman and Fielding, 2019) and is justified by the expected arrangement of proteins in the membrane (with hydrophobic helices in contact with the membrane and amphipathic helices forming a central hydrophilic pore), underwent the same equilibration and production run as Model A, with subsequent analysis presented in Figure 3 and Figure 4 on the 1 μs trajectory.

The RMSD analysis indicates that all five subunits remain stable on the microsecond timescale (Figure 3B), though for Model B this value is larger than for Model A. This does not surprise us, since for Model B we started from an unknown structure. To remove any doubts, we calculated the correlation among peptides and the root-mean-square-fluctuations (RMSF) (Supplementary Figure S5). Model A shows very low correlations and small RMSF, typical of a tight structure like the helix bundle that appears after equilibration (Figure 2 and Supplementary Figure S1/S2). On the other hand, Model B shows larger RMSF and more pronounced correlations, expected in a flexible system with an underlying structure with units interacting among them. We calculated the secondary structure on both the entire trajectories, Supplementary Figure S5-bis. As we can see, both models show only local (in time and space) rearrangements of their secondary structures. To note that Model B seems to have more stable helixes compared to Model A, having the latter more regions classified as Turn, especially for the short helixes.

Water molecules can now establish a stable central pore (Supplementary Figure S6A) free from hydrophobic residues, which are now positioned externally (Figure 3D). Moreover, calcium ions are more centrally located than before, and we observe a higher density of chloride ions inside the membrane layer (Figure 4), primarily on the supposed cytoplasmic side. The density of calcium near the luminal mouth (Figure 4) is significantly greater than the density of potassium ions. The presence of two glutamic residues on the N-terminal region contributes to this accumulation, which is more pronounced in Model B. On the other hand, chloride is more prevalent on the cytoplasmic side, especially in Model B and in the presence of calcium.

### *In silico* electrophysiology demonstrates model B allows Ca^2+^ permeation

In both models we examined the capabilities of water pathways to conduct ions by applying an external electric field. We used an external field value compatible with experimental conditions, ±100 mV, applied along a 1 μs trajectory after the previously described simulation of 1 µs When we observed low conductance in the initial trajectory, we extended the simulations to 2 µs Additionally, we assessed the stability of the oligomer when subjected to the external field by calculating the RMSD, as described previously.

Our findings are summarized in Table 1. We calculated the accumulation of charges over time, denoted as Q(t) (Aksimentiev and Schulten, 2005). By fitting Q(t) with a linear function, the ratio of the slope to the external potential represents the conductance. At a concentration of 150 mM KCl with no calcium, both models exhibited an exceedingly low conductance, approaching the limits of our resolution. This was especially noticeable during the second microsecond of simulation when charge equilibration was attained. Upon the addition of calcium, we observed a current only with Model B, measuring 600 pS. Also applying a −100 mV potential to Model B these results were confirmed. In the presence of KCl alone, the current was absent, but it exhibited some amplification in the presence of calcium (300 pS). Notably, the current for both voltages was primarily driven by the movement of chloride ions (Supplementary Figure S6B), possibly influenced by the force field used to represent calcium ions. In Model B, we assessed the stability of the subunits under the influence of the external electric field. Under positive voltage, the RMSD of all five units remained stable, while under negative voltage, one unit deviated by more than 5 Å from the others (Supplementary Figure S7). It is worth noting that, in the context of suggested protein E insertion into organelles, the positive voltage aligns with the membrane potential between the lumen (positive) and the cytosol.

**TABLE 1:** Ion conductance from microsecond simulations for Model A and Model B with different ion concentrations and a constant external potential of ±100 mV.

	+100 mV 150 mM KCl	−100 mV 150 mM KCl	+100 mV 50 mM KCl + CaCl_2_	−100 mV 50 mM KCl + CaCl_2_
Model A	1st μs: 10 pS		1st μs: 26 pS	
	2nd μs: 4 pS		2nd μs: 0.4 pS	
Model B	1st μs: 8 pS nd	1st μs: −2 pS nd	1st μs: 598 pS	1st μs: 313 pS
	2nd μs: −0.2 pS	2nd μs: 0.1 pS		

This observation is also consistent with the dipole moment of the long hydrophobic helices aligned with the membrane potential and the external electric field.

### Prediction of ion binding sites reveals C-terminal propensity to interact with calcium ions

Electrophysiological data and MD simulations suggest that the presence of calcium may enhance chloride permeation through the pore. To determine whether calcium ions bind to the protein, we conducted a comprehensive investigation of the entire structure of E-2. Notably, the C-terminal sequence displayed a higher propensity for binding calcium ions. The putative binding sites 45N-46I, 72D-69R, and 72D-73L exhibited the highest scores (see Table 2). Additionally, a single predicted binding site was identified at the beginning of the transmembrane domain (TMD) (15N-16S). It is intriguing to observe that the pair of glutamic acids at the N-terminus (residues 7 and 8) were not identified as optimal binding sites for calcium ions. In the pentameric structure, they remain in the aqueous environment at the luminal mouth, potentially contributing to the high concentration of calcium ions, as depicted in Figure 4.

**TABLE 2:** Ranking of predicted binding site for calcium along the entire structure of E2, using the specific software Metal Ion Binding 2 (MIB2).

Sites	Binding residues	Score
1	45N, 46I	2,858
2	72D, 73L	2,084
3	42Y, 45N, 46I, 48N	1,561
4	69R, 72D	1,534
5	45N, 46I, 48N	1,406
6	15N, 16S	1,262
7	64N, 65L	1,255
8	72D, 73L, 75V	1,226

This analysis was conducted based on the amino acid sequence of the envelope protein, as a complete tertiary structure is not available in the Protein Data Bank. The amino acid sequence is a shared element for both Model A and Model B. The results obtained suggest that the conformation adopted by the C-terminus in the Model B structure may promote the formation of specific active sites that interact with calcium ions present within the biological system. These interactions could potentially alter the pore diameter, facilitating ion passage. Furthermore, the results presented here align with the typical interaction of calcium ions with amino acids; indeed, Ca^2+^ predominantly interacts with carboxylated residues, such as aspartic and glutamic acid, as well as with any negative charges present in the side chains of asparagine, glutamine, serine, threonine, and tyrosine (Bennick et al., 1981; Pidcock and Moore, 2001; Kretsinger et al., 2023).

### Calcium and chloride transport

We conducted additional simulations using Model B of the protein E-2, which was embedded in 1,2-diphytanoyl-sn-glycero-3-phosphocholine (DiphPC) lipids under bi-ionic conditions, with the presence of 1M CaCl_2_, replicating the conditions of our electrophysiology experiments. For calcium, we utilized a recent multi-site parametrization capable of reproducing both thermodynamic and kinetic properties (Zhang et al., 2020; Liu et al., 2021).

The use of a high concentration of calcium was intended to identify the preferred region for calcium ion binding along the z-axis. We applied an external potential of 100 mV for 1 μs and observed a conductance of 72 pS, with a single event of calcium transport and several events of chloride transport, indicating a preference for conducting anions.

**FIGURE 5:**
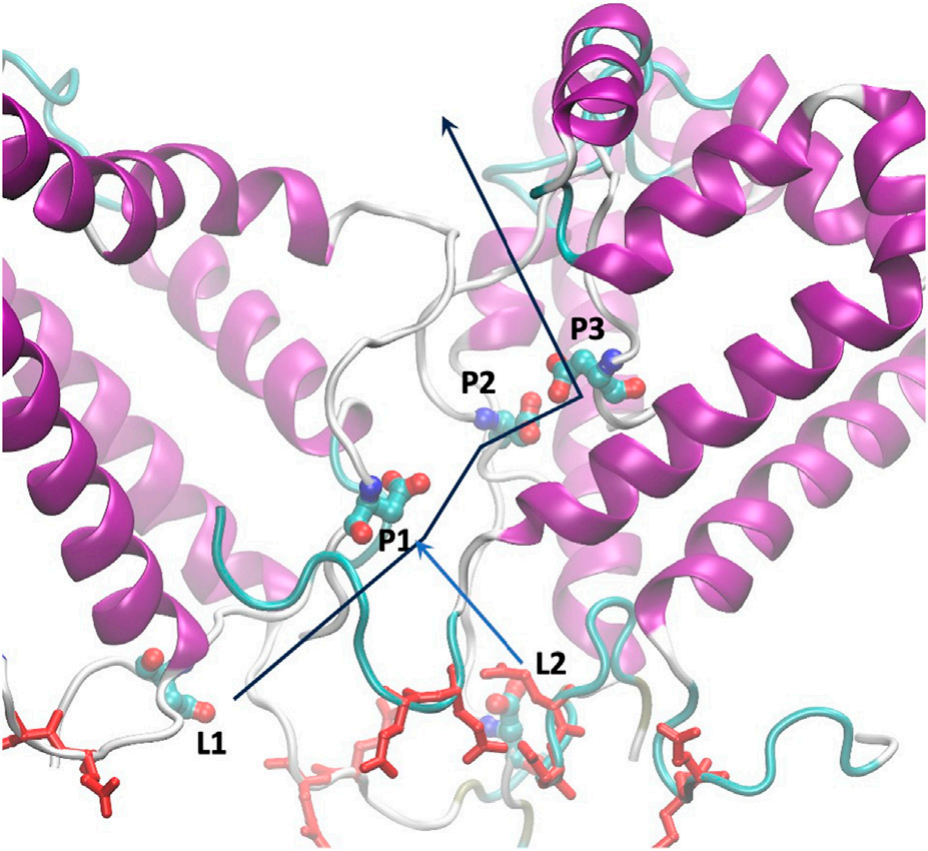
Arrangement of glutamic residues (red licorice) and aspartic residues at position 72 (CPK colored by atom name), labeled as in Tab4. The lines represent the path of calcium ions from the luminal side to the cytoplasm.

The analysis of single calcium ion permeation is of particular interest, see Figure 5. The calcium ion interacts with residue 72D, as predicted in our previous analysis, within the disordered C- terminus. As shown in Supplementary Figure S8, the aspartic acid at position 72 occupies different Z positions (depicted as orange spheres). The yellow calcium ion initially binds to one of the 72D residues in the central region of the pore, then a second 72D residue approaches and the ion is shared between the two aspartates. The second aspartate later moves upward with the calcium ion bound, and eventually, the ion moves upward as a second ion approaches the pair of aspartates. While we observed only a single passage event, this mechanism is reminiscent of the “knock-off” mechanism recently described for a calcium ion channel (Liu et al., 2021).

In accordance with Zhang et al. (Zhang et al., 2020), who achieved calcium transport by applying a high external voltage of 500 mV, we decided, after the 1 μs trajectory at 100 mV, to perform an *in silico* ramp experiment. This involved increasing the external field in steps of 100 mV every 100 ns, reaching a maximum of 400 mV before returning to 100 mV. This protocol was repeated three times, resulting in independent trajectories used to calculate the ionic currents.

**FIGURE 6:**
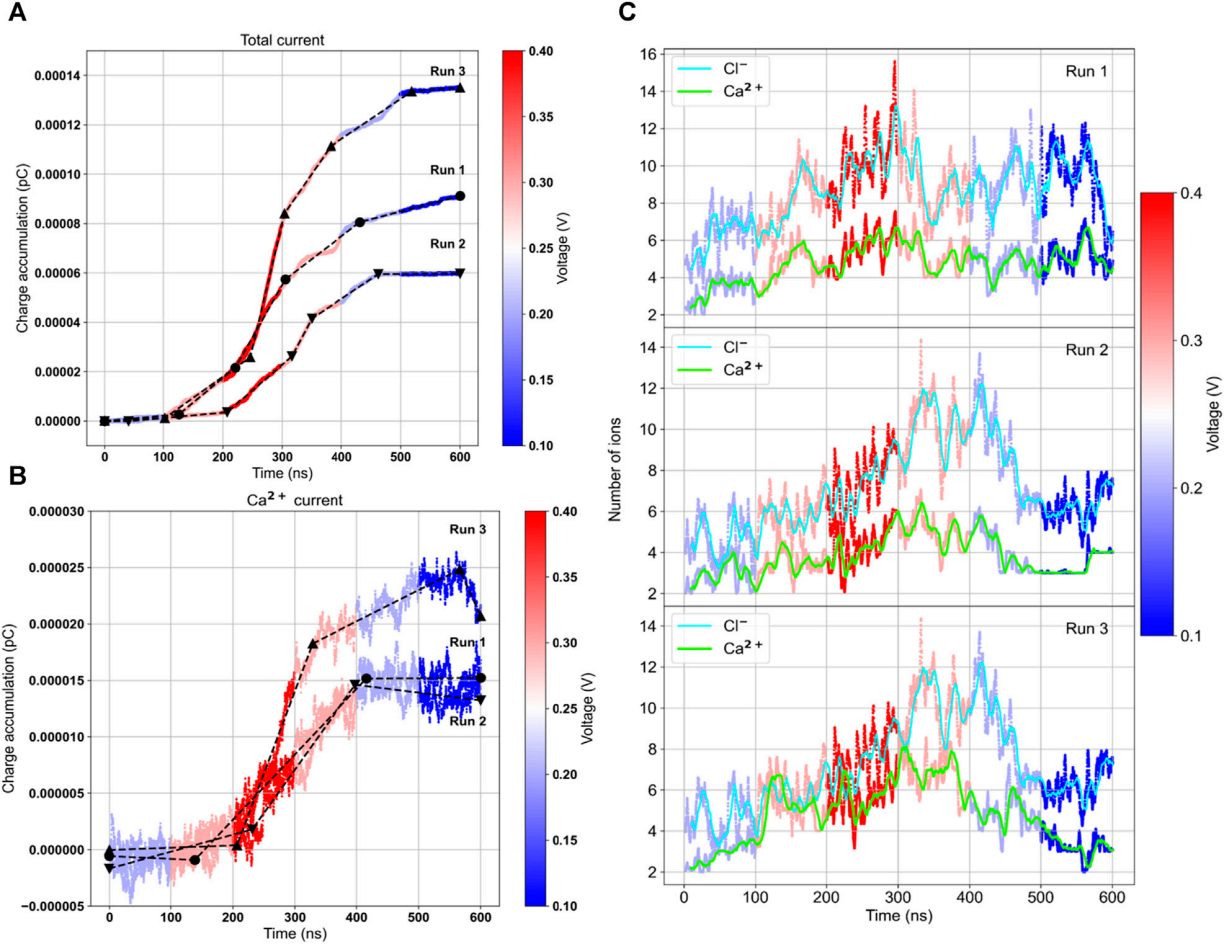
Ionic current and number of internal ions calculated on the three independent ramp voltage trajectories. Lines are colored according to the external voltage applied. Charge accumulation for the total current **(A)** and the calcium current **(B)**. The black lines represent the slope of the curves, proportional to the conductance. On the right the average number of chloride and calcium ions inside the central pore region **(C)**.

The results, as displayed in Table 3 and Figure 6, reveal several noteworthy observations. The three trajectories show significant heterogeneity with varying levels of conductance. Only when an applied voltage of 300 mV was used did we observe calcium ion transport. The conductance changes occurred after a delay following the change in voltage, requiring some relaxation to adapt to the new conditions. The conductance was higher after reaching the maximum voltage of 400 mV, and in RUN-2, we observed a remarkable calcium current even at 100 mV. This suggests that the channel’s activation is voltage-dependent. Under bi-ionic conditions within DiphPC membranes, there appears to be a preference for chloride transport, with the PCl^−^/PCa^2+^ ratio ranging from 1.7 to 6. Given the short simulations for each voltage, there is room to refine this number and compare it with our experimental data, which measured it at 2.6.

**TABLE 3:** Total conductance and calcium conductance on the three independent trajectories with bi-ionic conditions (1M CaCl_2_), as a function of the applied voltage.

Total current	RUN-1	RUN-2	RUN-3	Average
Applied Voltage				
**200** ** ** **mV**	0	0	0	0
**300** ** ** **mV**	513 pS	32 pS	467 pS	337 ± 217 pS
**400** ** ** **mV**	950 pS	430 pS	1,450 pS	943 ± 416 pS
**300** ** ** **mV**	490 pS	833 pS	1,067 pS	797 ± 237 pS
**200** ** ** **mV**	380 pS	600 pS	700 pS	560 ± 134 pS
**100** ** ** **mV**	630 pS	0	310 pS	313 ± 257 pS

Ca^2+^ Current	RUN-1	RUN-2	RUN-3	Average
Applied Voltage				
**200** ** ** **mV**	0	0	0	0
**300** ** ** **mV**	102 pS	85 pS	74 pS	88 ± 12 pS
**400** ** ** **mV**	136 pS	190 pS	356 pS	227 ± 90 pS
**300** ** ** **mV**	106 pS	280 pS	140 pS	176 ± 70 pS
**200** ** ** **mV**	6 pS	0	160 pS	55 ± 74 pS
**100** ** ** **mV**	10 pS	0	0	3 ± 5 pS

We also calculated the number of ions inside the internal water pore within a range of [-15:+15] Å. Interestingly, there is a correlation between the number of internal calcium ions and the current. When there are more than 4 calcium ions inside the pore, achieved by increasing the voltage, a current is observed. In RUN-2, the current remained low until 400 mV when the average number of ions exceeded 6. In RUN-3, at the end of the trajectory, the number of calcium ions dropped to 2, and the current ceased. Thus, it appears that the bottleneck for transport is the internal occupancy of the water pore.

As previously mentioned, there are five aspartic acids facing the water pore, situated in the C- terminus, an unstructured region. Their variable Z positions at high voltage result in an average of more than 4 internal ions, with all aspartic acid carboxylic groups being saturated. The entry of any new ion promotes the exit of an internal ion, reminiscent of a similar mechanism suggested for other calcium channels (Tsien et al., 1987). We recently observed a similar behavior with the sodium channel hTPC2 (Milenkovic et al., 2021), where the occupancy of the water central cavity decreased the transport barrier, facilitating ion passage via the knock-off mechanism.

Lastly, we briefly opened the pore with a short 400 mV simulation (50 ns) and performed 200 ns simulations at lower voltages. Calcium current was observed in three independent trajectories only at 300 mV, yielding 13, 10, and 18 permeation events (Supplementary Figure S9), corresponding to 104, 80, and 144 pS, respectively. At 200 mV, there were 4, 5, and 0 events, and in a single 100 mV simulation, no events were observed.

To analyze quantitatively calcium transport, we calculated the survival probability along the simulations at 300 mV (N frames):

$$S\left(t\right)=\frac{1}{N}\sum_{n=1}^{N}\sum P_{j}\left(t_{n},t\right)$$

summing over all calcium ions j, being Pj = 1 or 0 respectively when the ions is bound or not to the selected residues, in this case the list of aspartic and glutamic acid. This function quantifies the binding/unbinding kinetics of small ligands to residues and can be fitted via a sum of exponential functions:

$$S\left(t\right)≅n_{1}e^{-t/{\tau au}_{1}}+n_{2}e^{-t/{\tau au}_{2}}+n_{3}e^{-t/{\tau au}_{3}}$$

where the coefficient ni represent the number of ions/fraction of ions that bind for a time Taui^.^ This approach allowed us to examine the kinetics of water molecules on the protein surface (Sterpone et al., 2001) and the unbinding of oxygen/water from a cavity (Bui et al., 2023). The function S(0) has a special meaning: it represents the average number of ions bound to the selected residues, that approximate the occupancy in Table 4.

**TABLE 4:** Parameter analysis of the survival function for calcium binding *versus* each 72D residue (L1, L2, P1, P2, P3) and the five together (ALL). In the last column we reported the cumulative survival probability for calcium *versus* the 10 GLU residues of the N-terminus.

	**L1**	**L2**	**P1**	**P2**	**P3**	**All**	**10 GLUs**
**Z Position ± STD**	**−19.3 Å** ± **0.9**	**−19 Å** ± **1.5**	**−7.9Å** ± **1.3**	**−0.38Å** ± **0.8**	**−0.25Å** ± **0.8**		**[-17: 25]Å**
**Occupancy ± STD**	0.6 ± 0.6	0.4 ± 0.5	1.1 ± 0.6	1.5 ± 0.6	1.5 ± 0.7	4.4 ± 1.2	7.5 ± 1.7
**Exponential Fitting: S(t) = n**_**1**_ **exp(-t/Tau**_**1**_ **) + n**_**2**_ **exp(-t/Tau**_**2**_ **) + n**_**3**_ **exp(-t/Tau**_**3**_ **)**							
**Tau**_**1**_	0.81 ns ± 0.01	0.314 ns ± 0.002	0.85 ns ± 0.02	-	1.00 ns ± 0.01	0.40 ns ± 0.01	0.407 ns ± 0.005
**n**_**1**_	0.575 ± 0.003	0.410 ± 0.01	0.628 ± 005	-	0.68 ± 0.01	1.27 ± 0.02	5.72 ± 0.04
**Tau**_**2**_	-	-	8.2 ns ± 0.1	2.36 ns ± 0.03	6.2 ns ± 0.1	2.44 ns ± 0.04	2.59 ns ± 0.05
**n**_**2**_	-	-	0.435 ± 0.003	0.877 ± 0.007	0.76 ± 0.01	1.32 ± 0.01	1.54 ± 0.04
**Tau**_**3**_	-	-	-	15.2 ns ± 0.2	-	15.28 ns ± 0.04	-
**n**_**3**_	-	-	-	0.517 ± 0.007	-	1.80 ± 0.01	-
**(n**_**1**_ **+n**_**2**_ **+n**_**3**_ **)/Occupancy**	91%	95%	97%	96%	97%	100%	97%

As mentioned earlier, the 72D residues, located on the C-terminal and within a flexible region, exhibit varied spatial positions compared to what might be expected in an ordered symmetric system. We categorized the residues based on their Z positions along the pore (see Table 4). L1 and L2 represent the two outermost residues facing the lumen, while P1, P2, and P3 represent the three inside the pore, with P1 closest to the lumen and P3 closest to the cytoplasm.

We initially calculated the probability of calcium binding to each individual residue. As shown in Table 4, the internal residues can bind more than one calcium ion, with P2 and P3 reaching an average occupancy of 1.5. In contrast, L1 and L2 exhibited an occupancy of around 0.6. Subsequently, we calculated the kinetics of unbinding by determining the survival probabilities, from which we derived residence times before escape. The unbinding process follows a random process with a Poisson distribution. It is worth noting that for some residues, we observed more than one escape time, and the survival function could be fitted with more than one exponential.

Both L1 and L2 exhibited calcium unbinding with a single exponential decay, with residence times of less than 1 ns P1 and P3 displayed similar fast kinetics and additional unbinding events with residence times between 5 and 10 ns In contrast, the central residue, P2, displayed a longer fast residence time of 2.4 ns and an extremely long slow residence time of 15.2 ns When considering the occupancy of all five residues together, an occupancy of 4.4 out of 5, or 90% single occupancy, was observed, with three characteristic times for unbinding. Two of these times corresponded to those found for P2, and the third was less than 1 ns. Supplementary Figure S9 depicts the trajectories along the membrane axis followed by all permeating calcium ions.

## Discussion and conclusion

The protein E is the least known among the SARS-CoV-2 structural proteins, although its ability to form pores at the “ERGIC” level has been largely confirmed (Venkatagopalan et al., 2015). Indeed, so many unanswered questions remain about the ion transport through the channel and the three-dimensional arrangement of protein oligomers within the membrane environment. Herein, we proposed a new arrangement of the pentameric structure of the protein E-2 (Supplementary Figure S3), named Model B, which we compared with the model derived from NMR experiments, Model A (Surya et al., 2018). Only the new arrangement predicts the presence of water in the membrane so that a stable and open pore is formed on the microsecond time scale (Supplementary Figure S6). This pore-forming pentameric structure can transport ions, and our positive potential, that provides the highest current, corresponds to having the same electric field between the lumen and the cytosol as in the cell. In this case the dipole of long helices is aligned to the electric field. Further, from the analysis of the RMSD, we noticed that at positive voltage the pentameric structure is stable (Supplementary Figure S7), as for the trajectory without electric field (Figure 3B). On the other hand, at negative voltage one of the units deviates by more than 5 Å from the mean structure (Supplementary Figure S7).

According to our simulations, calcium acts as an amplifier of the ionic current, Table 1. Interestingly, calcium accumulates more in the supposed lumen part, dur to the presence of a pair of glutamic acid residues. Experimentally, we could measure an ion current in an electrophysiology apparatus only in the presence of calcium. Thus, at the moment, this new arrangement of E-2 and its N-terminal insertion in the membrane provides the most stable structure, given the conditions imposed by organelles, namely, the membrane potential and a high calcium concentration on the lumen side (Figures 4B).

Using a more sophisticated force field for calcium ions, with 7 sites (Zhang et al., 2020), we were able to quantify by simulations its voltage gating behavior and the conductance (Figure 6). Though the structure of this pentameric pore-forming peptide does not present any regularity compared to ion channels, we were able to derive some interesting and rational rules for permeation. The selectivity for calcium *versus* potassium is probably due to the sequence, since the pair of glutamic acids per peptide are exposed at the luminal mouth in the N-terminus (Figure 5). This provides a high density of calcium ions that would prevent, according to the charge/space competition model (Boda et al., 2000), the arrival of potassium (Figures 4B).

The bottleneck for transport appears to be the saturation of the central water pore. There, we have 5 aspartic acids of the C-terminus (position 72), and because of the flexibility of their side chain, they occupy different regions along the diffusion axis Z, on average in the range [-20:0] Å, (Figure 5). Thus, only when almost all aspartic acids are occupied (4.4 calcium ions inside, on average, or 90% of occupancy of the 5 residues), we could see a current increase. To note that the 90% occupancy is on the 5 residues seen as a unique system. If we examine the occupancy of each single residue, we see two residues in the cavity, P2 and P3, having average occupancy around 1.5, meaning that for half of time they have double occupancy (Table 4).

Within this condition, the carboxylic groups of aspartic acids are saturated by calcium ions, and new ions injected in the pore push an internal calcium ion to exit, reminding the knock-off mechanism also proposed very recently for other calcium channels (Schackert et al., 2023). In particular, the residue at position P2 has the highest residence time for calcium, providing a bridge between position P1 and P3, Figure 5.

Our simulations with the external electric field suggest that the pore is anion selective, contrary to what has been reported to date in literature (Trobec, 2021). Experimentally, in order to characterize the E-2 protein, we reconstructed the lipid system at pH 6, in order to mimic the ERGIC environment, which has a slightly acidic pH, normally between 6 and 7 (Medeiros-Silva et al., 2022). Accordingly, PLB electrophysiological recordings confirm recombinant E-2 channel is slightly anion selective (P_cl_/P_ca_ = 2.6) when reconstituted in neutral phosphatidylcholine membranes. However, negatively charged lipid bilayers made of DiphPC and DiphPG in ratio 4:1 switched the pore preference towards cations, as revealed by the reversal potential of +11.47 ± 0.40 mV that corresponds to a P_cl_/P_ca_ of 0.9. These interesting results could be explained by the modulation of viroporins selectivity by environmental conditions such as the pH, the composition of the membrane and the ion concentrations already proposed for SARS-CoV E protein (Nieto-Torres et al., 2015; Verdiá-Báguena et al., 2012). These data indicate that lipid charge would largely influence the channel preference for Ca^2+^, showing that under conditions mimicking the ERGIC/Golgi environment, E-2 protein would display a mild selectivity for Ca^2+^. This selectivity observed for the calcium ions may lead to dis-homeostasis phenomena related to the concentration of the same ion in the cytosol. Indeed, the concentration of calcium ions within the ERGIC complex is much higher than that observed in the cytoplasm (Medeiros-Silva et al., 2022). Once released, calcium could act as a second messenger by altering cellular pathways, as suggested in previous studies of the envelope protein from SARS-CoV (Nieto-Torres et al., 2015).

In conclusion, we propose a new arrangement of the protein E able to form a stable water pore, matching electrostatically the organelles environment. Only within this arrangement ionic current of both chloride and calcium ions was demonstrated, even if future investigations are calling for a more realistic treatment with longer simulations, eventually with the double membrane system (Kutzner et al., 2011) and/or a different composition of the membrane. Site directed mutagenesis will also be a valuable tool in future to explore the mechanism of calcium conductance.

Our results revolutionize the previously described three-dimensional structure of E-2 from NMR and provide a brand-new contribution in deciphering the molecular mechanisms of channel functioning. This is not surprising when dealing with peptides-forming pore, as also revealed by a recent contribution where the bundle obtained with X-ray diffraction was not able to conduct ions (Scott et al., 2021). These data are essential and propaedeutic for understanding the biological cycle of SARS-CoV-2 and its viral family and lay the basis for the design and development of E-2-targeted drugs aimed at contrasting future outbreaks and potentially consequent pandemics.

## Materials and methods

### Cloning and expression of the envelope gene from SARS-CoV-2

RNA extraction was performed, as previously described (Musso et al., 2020; Musso et al., 2021), employing QIAamp Viral RNA Mini Kit (Cat. No. 52906, Qiagen, Hilden, Germany) with some modifications based on the complex features of the starting samples. For the synthesis of the recombinant protein, the sample showing the lowest number of SNPs in the E gene portion of interest was selected through an accurate analysis of Next-Generation Sequencing data from about 200 SARS-CoV- 2 positive samples. E gene was amplified by PCR from the cDNA of a COVID-19 positive patient using the following primer pair: E2Fw, 5′- GTT​TAA​CTT​TAA​GAA​GGA​GAT​ATA​CAT​ATG​TAC​TCA​TTC​GTT​TCG​GAA​GAG​ACA​G G-3’; E2Rev 5′- TCA​GTG​GTG​GTG​GTG​GTG​GTG​CTC​GAG​GAC​CAG​AAG​ATC​AGG​AAC​TCT​AGA​G-3’. Subsequently, cloning of E2 into the pET21b vector (Novagen) was achieved with another primer pair: E2-1Fw 5′- GTT​TAA​CTT​TAA​GAA​GGA​GAT​ATA​CAT​ATG​TAC​TCA​TTC​GTT​TCG​GAA​GAG​ACA​G G-3’; E2-1Rev 5′- TCA​GTG​GTG​GTG​GTG​GTG​GTG​CTC​GAG​GAC​CAG​AAG​ATC​AGG​AAC​TCT​AGA​G-3′ by means of the NEBuilder HiFi DNA Assembly Master Mix (New England BioLabs) to produce a recombinant protein containing a C-terminal His6-tag. Protein expression was induced in *Escherichia coli* BL21 by addition of 0.8 mM Isopropyl-β-D-thiogalactopyranoside (IPTG) at 20°C for 16 h. After cell lysis with Lysis Buffer (50 mM Trizma Base, 100 mM NaCl, 1% Triton X-100, 0.1 mg/mL lysozyme, 10 mM imidazole, 6M urea, pH 8.0), protein purification was achieved using Ni-NTA affinity cromatography under non-denaturing conditions according to the manufacturer’s instructions. Briefly, 20 mL of lysed sample were loaded onto a Ni-NTA agarose (Thermo Fisher Scientific, Waltham, MA, United States) packed column, previously equilibrated with Lysis Buffer. The column was then washed with 6 mL of Wash Buffer (50 mM Trizma, 100 mM NaCl, 0.1% LDAO, 20 mM imidazole, 3M urea, pH 7) and E2 protein was eluted with 800 μL of Eluition Buffer (50 mM Trizma, 100 mM NaCl, 0.1% LDAO, 250 mM imidazole, pH 7). Protein purification was assessed by western blot analysis with His6-tag antibody (1:1,000, 11922416001 Roche) and IRDye^®^ 800CW Goat anti-mouse IgG (1:30000, 926-322210, Li-Cor Biosciences) using the Odyssey CLx imaging system (Li- Cor Biosciences)

### Electrophysiological recordings

Electrophysiological experiments were carried out using the Planar Lipid Bilayer (PLB) technique according to (Checchetto et al., 2014; Reina et al., 2016; Conti Nibali et al., 2021). In brief, 1% DiphPC (Avanti Polar Lipids, Alabaster, AL) in n-decane was used for painting bilayer membranes with an approximate 110–150 pF capacity on a 200 μm aperture of a Delrin cuvette (Warner Instruments). Single channel recordings were carried out in symmetrical 1M CaCl_2_, 10 mM HEPES, pH 6 and at 100 mV applied following addition of 50 ng of the purified protein to the *cis* side of the chamber. Voltage-dependence and ion selectivity were investigated in 0.05 M–1 M asymmetric CaCl_2_ gradient using a 10 mHz triangular voltage wave from −100 to +200 mV. Current traces were recorded using the BC-535 Bilayer Clamp amplifier (Warner instruments) and data were digitally filtered using a low-pass-filter at 300 Hz and a sampling frequency of 10 kHz with the Axon Digidata 1,550 Acquisition System (Warner Instruments). Pore conductance (G) was calculated as the ratio of channel current (I) to applied voltage (V). At least three independent experiments were repeated for each condition.

### Molecular dynamics simulations

For Molecular Dynamics simulations we prepared two different structural systems. The first one (Model A) was obtained by homology modelling (using Modeller 10.1) (Webb and Sali, 2016) employing the E protein PDB files as initial coordinates (PDB ID: 5 × 29) and adding the specific missing residues. The other structure (Model B) was obtained after re-orientation of each monomeric domain by a rotation of 180^o^ with respect to the membrane axis.

The CHARMM-GUI web server (available for free after registration at https://www.charmm-gui.org) (Jo et al., 2008; Lee et al., 2019) was used to prepare both cell-like systems. First of all, the rebuilt protein was inserted in a pre-equilibrated lipid bilayer system consisting of 75 DOPC (1,2-Dioleoyl-sn-glycero-3- phosphocoline, zwitterionic), 25 DOPS (1,2-Dioleoyl-sn-glycero-3-phospho-L-serine, negatively charged) and 25 DOPE (1,2-Dioleoyl-sn-glycero-3-phosphoethanolamine, zwitterionic) molecules, for both the upperleaflet and the lowerleaflet. Lipids form an xy-plane with initial size of 105 Å × 105 Å. The bilayer-protein complex was then immersed in an explicit water solution (TIP3P model) with several ionic concentrations: 150 mM KCl, 50 mM KCl +50 mM CaCl_2_. The related files for energy minimization, equilibration and production were directly provided by CHARMM-GUI, and the GROMACS version 2022.4 was used to run them using the CHARMM 36 m force field. A short 2 ns equilibration was performed applying restraints to the protein, followed by a long production run for a total of 1us in the NPT ensemble, the latter used to evaluate system stability. Pressure and temperature were held constant at 1 atm and 310 K using the Berendsen coupling scheme during equilibration. Instead for the production run, the Parrinello-Rahman coupling and the Verlet scheme were employed. Moreover, electrostatic interactions were treated with the smooth particle-mesh Ewald technique. Finally, MD simulations were performed in the NPT ensemble, by the application of an external electric field (E) in the direction of z-axis (Lz). To do this, a specific transmembrane voltage (V) has been chosen, knowing that V = EZ Lz. In this way, each charged particle feels about a force FZ = qEZ (Aksimentiev and Schulten, 2005). In the set of simulations with the DiphPC membrane and 1M CaCl_2_, as in experiments, we employed the recently developed 7 multisite model for calcium ions.

### Ca^2+^ binding site predictions

To evaluate the presence of putative Ca^2+^ binding sites along the entire structure of E-2, the software MIB2: Metal Ion-Binding site prediction and modelling server (available at http://bioinfo.cmu.edu.tw/MIB2/) was used. As no complete 3D structure of the envelope protein is available, this software allows the direct reconstruction of the tertiary structure from the primary sequence by homology modelling. All structures obtained are analysed without the use of constraints. Binding site evaluation is performed using the (PS) method as described in (Lu et al. (2022))

### Survival function

Since the calcium ions have a preference of interaction with acidic residues, we calculated the time-scale of this interaction to investigate the mechanism of transport. Based on the definition of survival function:

$$S\left(t\right)=\frac{1}{N}\sum_{n=1}^{N}\sum P_{j}\left(t_{n},t\right)$$

P_j_ is 1 or 0 when the calcium is at the distance of less or more than a given cutoff distance from any of the atom of the selected residues. The cutoff distance is defined as the sum of VDW radii of the calcium ion, 1.768 Å, and each other atom of the selected residues. If we imagine the binding/unbinding as a Poisson process, we can approximate the S(t) as a sum of exponential functions. In the past for the fastest processes, less than 1 picosecond, we used stretched potentials (Sterpone et al., 2001). However, in this case we are interested to much longer time scale and the frames of the trajectory are saved every 100 ps, thus our S(t) is well approximated as the sum of three simple exponentials:

$$S\left(t\right)≅n_{1}e^{-t/{\tau au}_{1}}+n_{2}e^{-t/{\tau au}_{2}}+n_{3}e^{-t/{\tau au}_{3}}$$

The S(0) represents the number of ions bound on average along the trajectory. This number might differ, only slightly, from the sum of ni, being the sum an approximation to the true value of S(0).

We calculated S(t) singularly for each aspartic acid at position 72 in monomers (labeled as L1, L2, P1, P2, P3) and we selected all these residues together (“ALL” in Table 4). Eventually we calculated S(t) for all 10 glutamic residues in the N-termini (“10 GLUs” in Table 4).

## Data Availability

The raw data supporting the conclusion of this article will be made available by the authors, without undue reservation.

## Abbreviations

SARS-CoV-2, Severe Acute Respiratory Syndrome coronavirus 2; E-2, Envelope protein from SARS-CoV-2; S, Spike protein; N, Nucleocapsid protein; M, Membrane protein; E, Envelope protein; PLB, Planar Lipid Bilayer; MD, Molecular Dynamics; DiphPC, 1,2-Diphytanoyl-3-phosphocholine; DiphPG, 1,2-diphytanoyl-sn-glycero-3-phospho-(1′-rac-glycerol) ; DOPS, 1,2-Dioleoyl-sn-glycero-3-phospho-L-serine; DOPC, 1,2-Dioleoyl-sn-glycero-3-phosphocoline; DOPE, 1,2-Dioleoyl-sn-glycero-3-phosphoethanolamine; RMSD, Root-Mean-Square Deviation.
