# Supplementary material for: Electrophysiological properties and structural prediction of the SARS-CoV-2 viroprotein E
Source: Front Mol Biosci. 2024 Mar 28;11:1334819. doi: 10.3389/fmolb.2024.1334819 (PMC11007222; doi:10.3389/fmolb.2024.1334819)
Supplement: Supplementary file 1 [file DataSheet1.PDF]

SUPPLEMENTARY INFORMATION

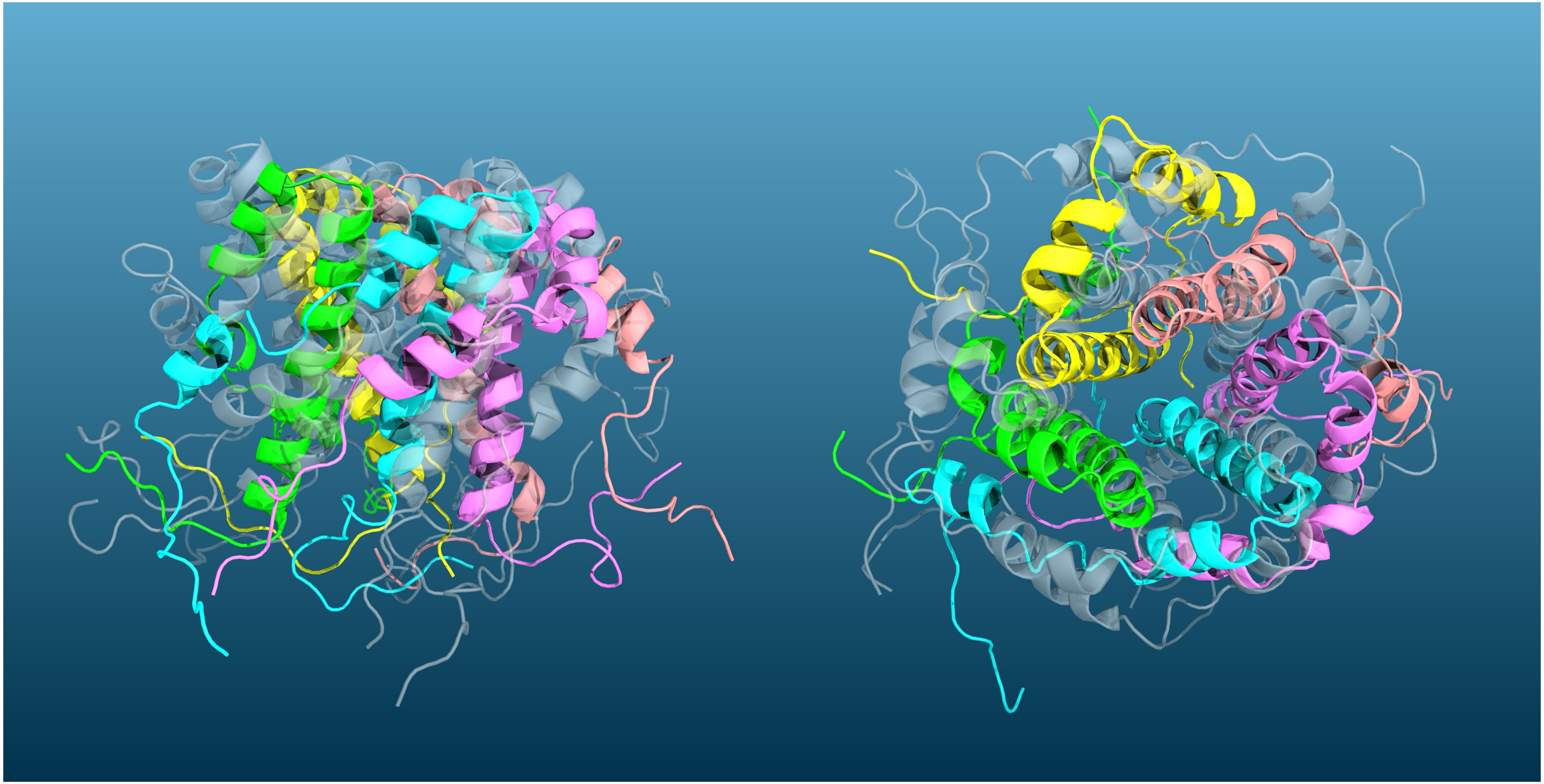

Figure S1: Side and top view of helixes arrangements in Model A at the beginning (gray) and after the 1us production run (colored).

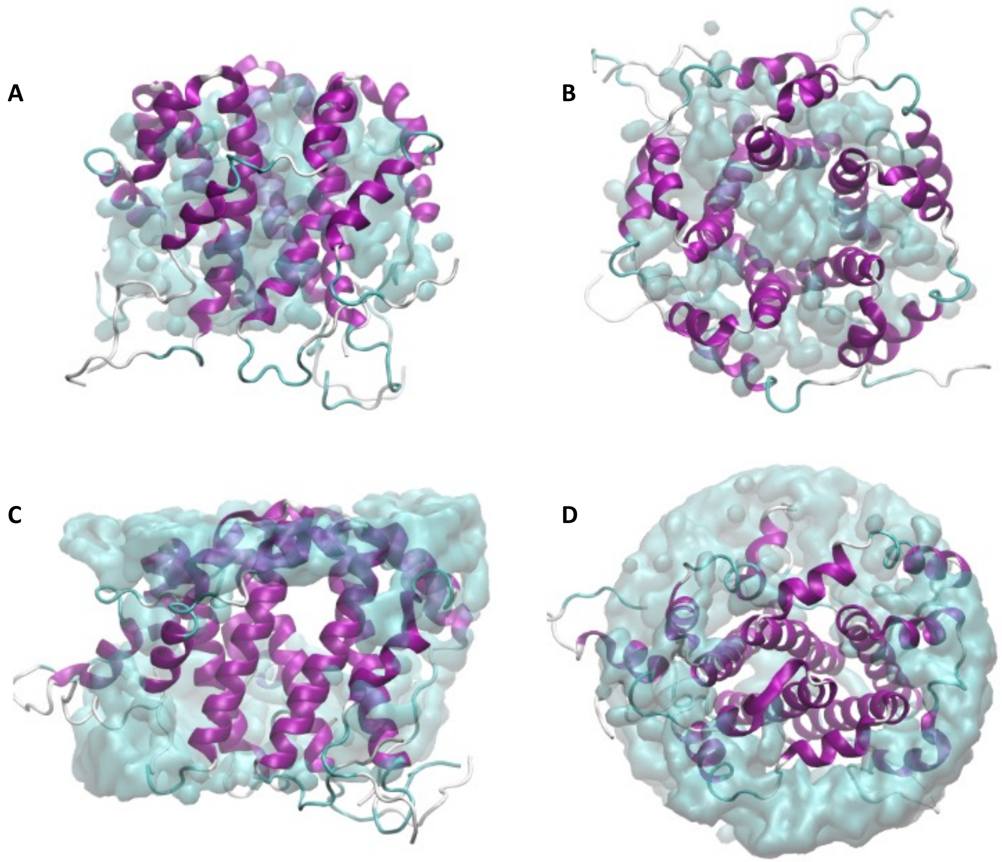

Figure S2: Pentameric structure of E2 Model A before (top) and after (bottom) the release of constraints on alpha carbons. Water molecules in contact with the protein are represented as a cyan surface**. (A,C)** side view**. (B,D)**: top view

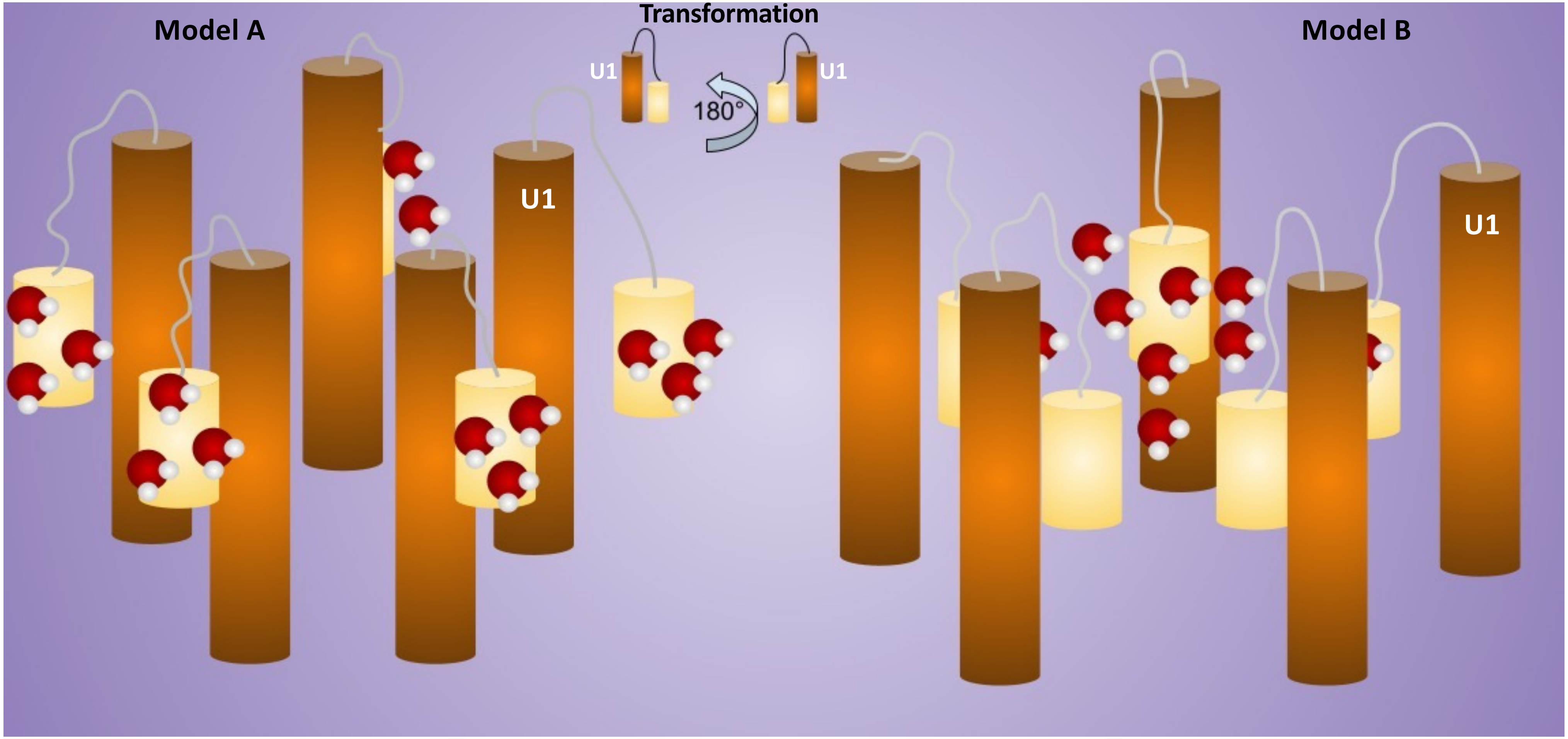

Figure S3: Scheme of the initial arrangement of helices in Model A (left) versus Model B (right), obtained with a rotation of 180 degrees applied to each monomer with respect to the axis of the membrane. In Model B the amphiphilic short helixes form a bundle and thus a stable water pore. We labeled a unit (U1) to compare its relative position after the rotation.

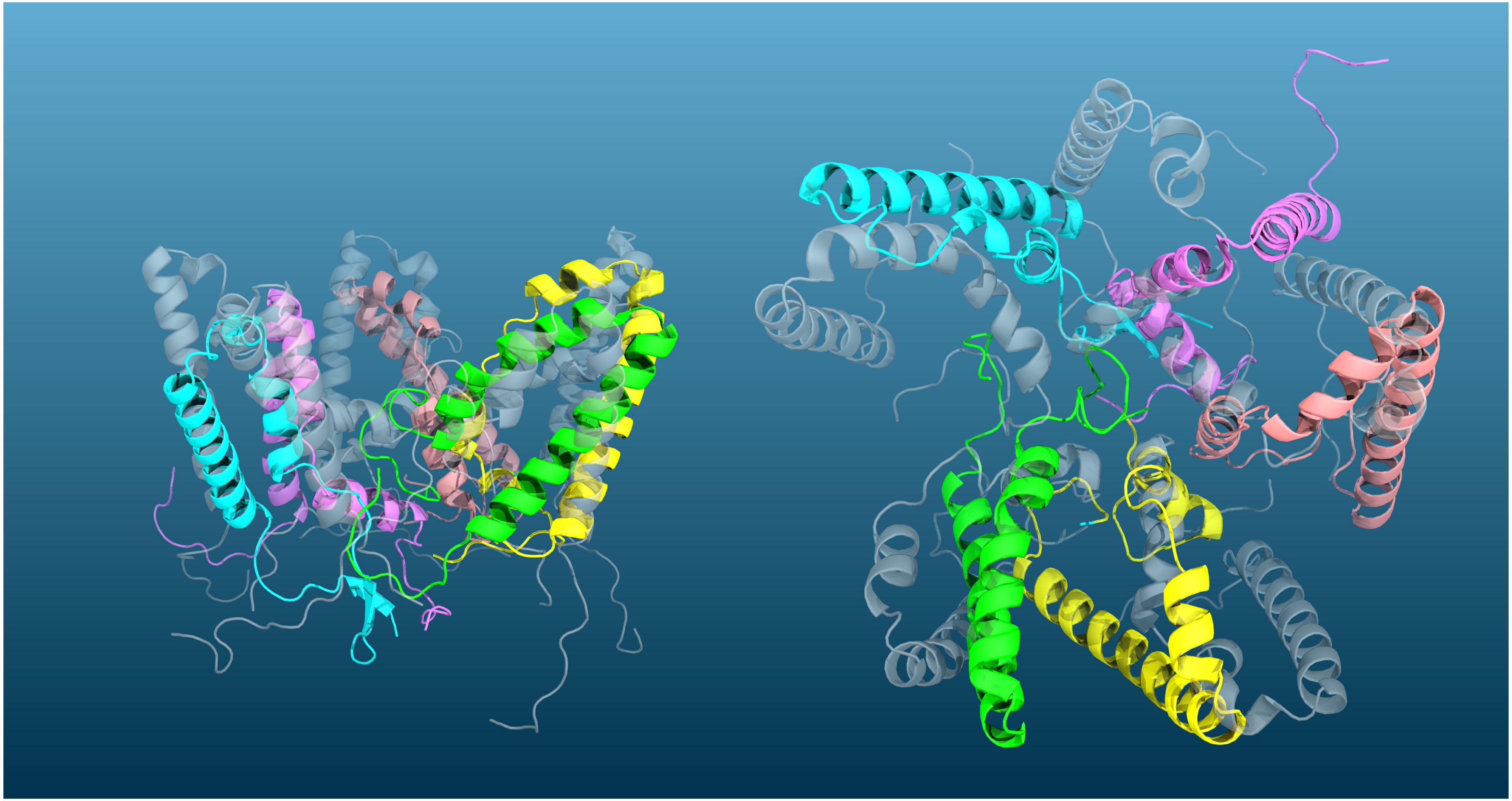

Figure S4: Side and top view of helixes arrangements in Model B at the beginning (gray) and after the 1us production run (colored).

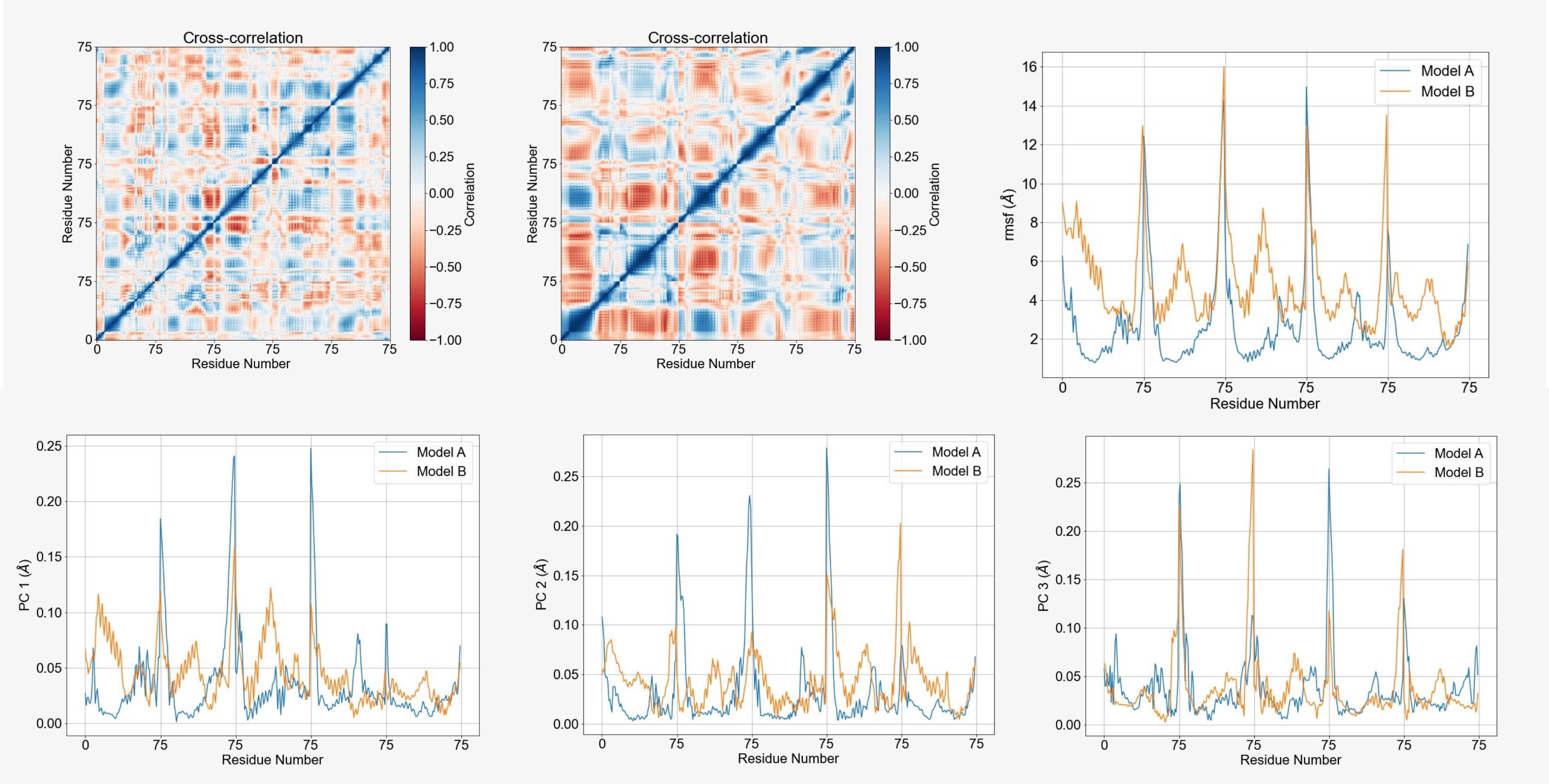

Figure S5: Correlation matrix for Model A (left) and Model B (right) and rmsf calculated on all atoms and selectively for the atoms of the first three principal component vectors.

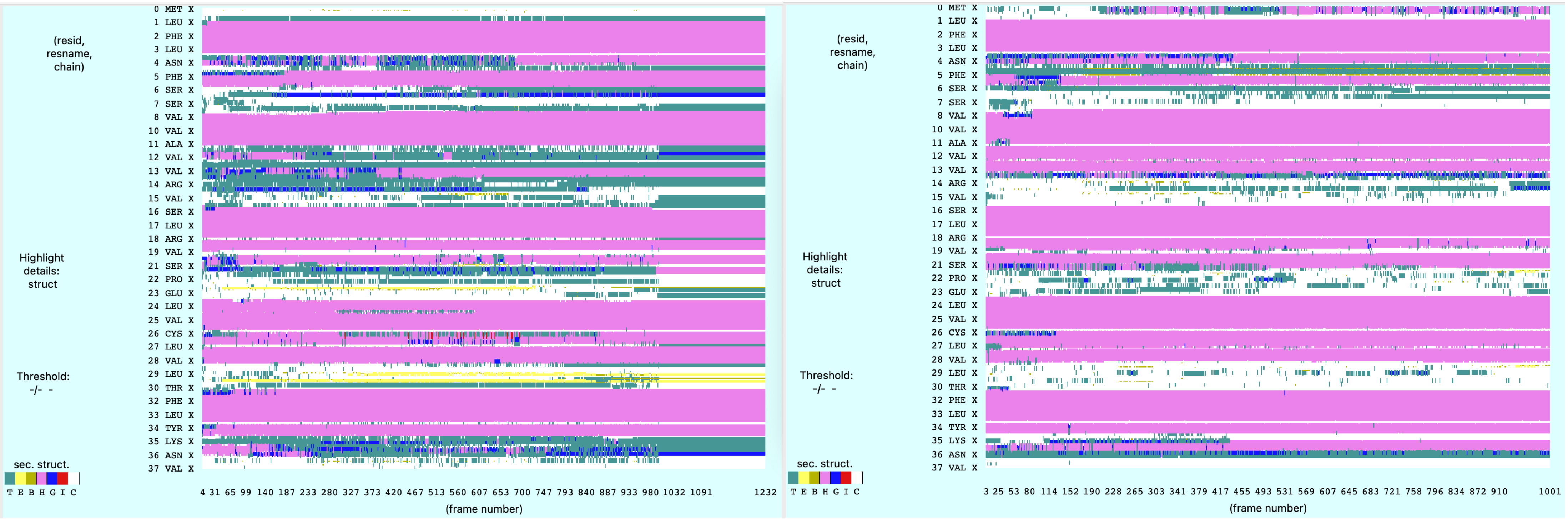

Figure S5-bis: Timeline of the secondary structure calculated on the entire 1 μs trajectories, left model A, right model B. Legenda of secondary structure: T = Turn; E = Extended conformation; B = Isolated bridge; H = Alpha helix; G = 3-10 helix; I = Pi helix; C = Coil, none of the above.

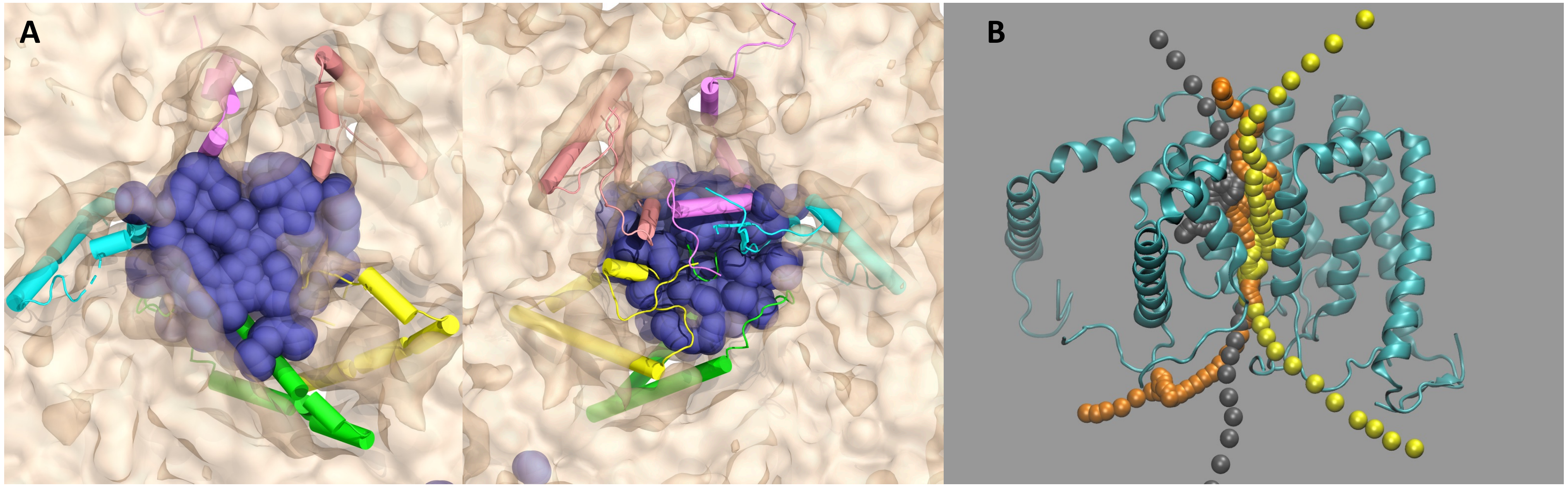

Fig. S6. (**A**): top/down view of the oligomeric structure embedded in the membrane and with the central pore occupied by water after 1 μs simulations, Model B. The membrane is represented in light yellow, while the protein monomers are colored differently and water molecules are in blu. (**B**): trajectories of three chloride ions when diffusing inside the water pore of Model B upon application of +100mV (electric field is directed in the up direction), in a solution of 50 mM KCL and 50 mM $CaCl_2$

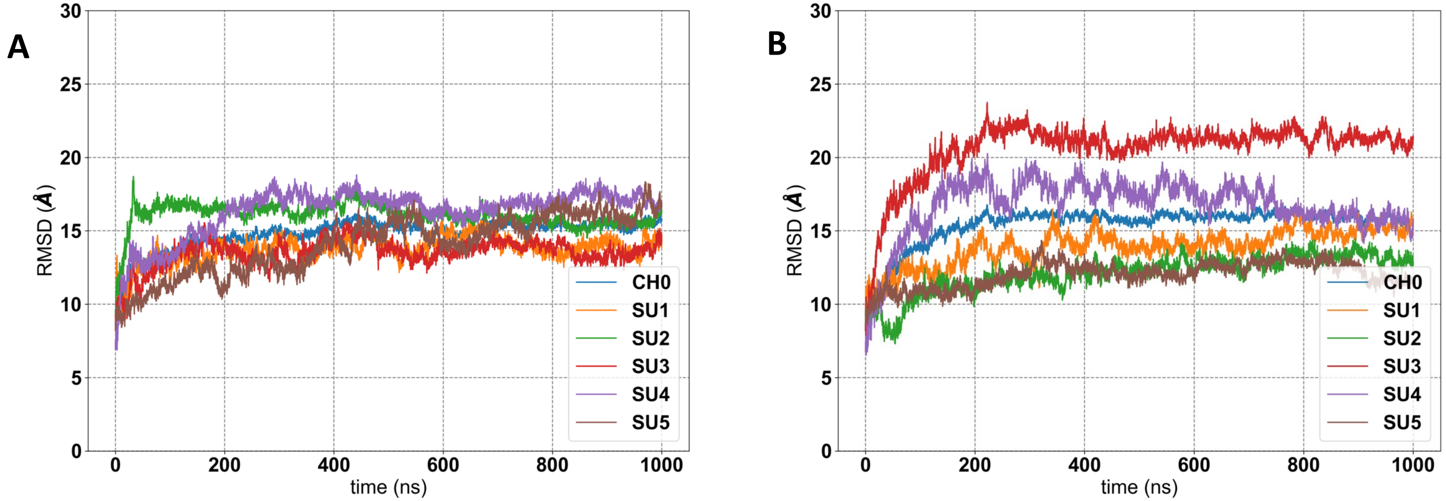

Fig. S7 Model B: Root-Mean-Square-Deviation (RMSD) of each monomer (SU 1-5) and the entire protein (CH0) from the initial configuration upon application of the external electric field of +100mV (**A**) and -100mV (**B**)

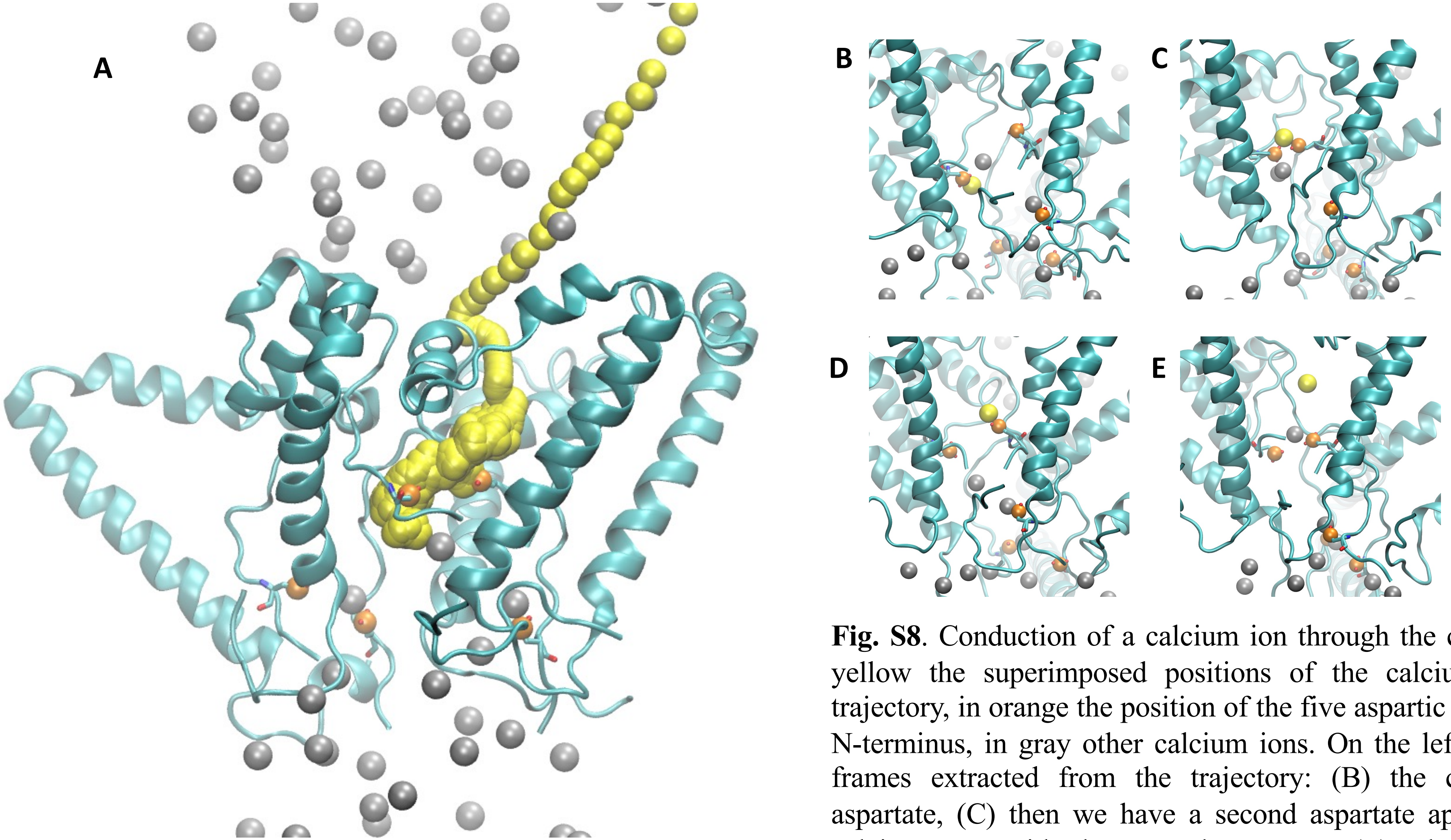

Fig. S8. Conduction of a calcium ion through the central pore. (**A**) In yellow the superimposed positions of the calcium ion during the trajectory, in orange the position of the five aspartic acid residues of the N-terminus, in gray other calcium ions. On the left the most relevant frames extracted from the trajectory: (B) the calcium binds one aspartate, (C) then we have a second aspartate approaching, (D) the calcium stay with the second aspartate, (E) when another calcium approaches the calcium ion moves up. The transport is favoured by the flexibility of aspartic acids in the N-terminus

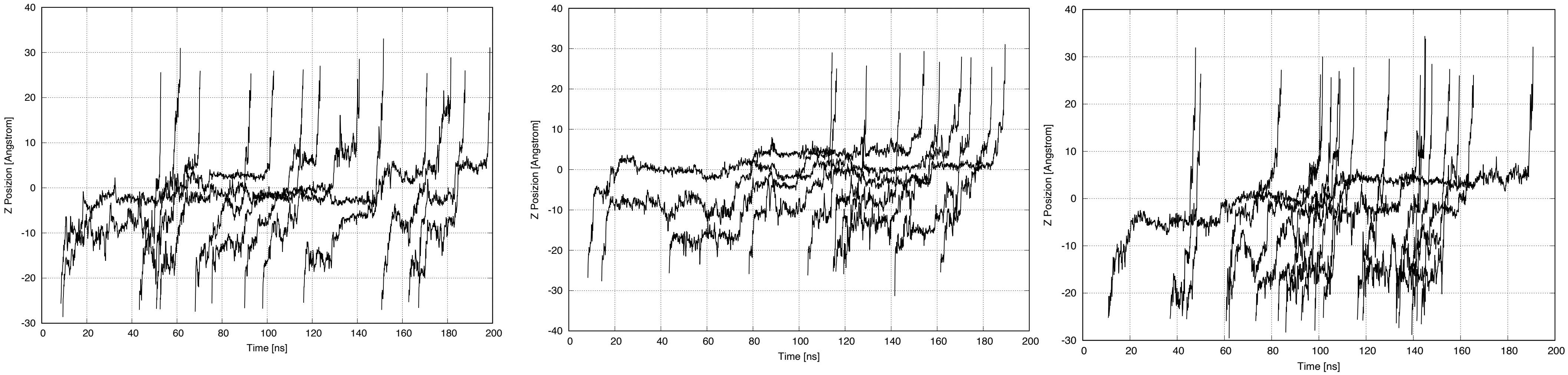

Fig. S9. Trajectories of calcium ions along Z during the 3x200 ns simulations with an external electric field of 300 mV
